# Report of longan witches’ broom-associated virus in lychee imported from China

**DOI:** 10.1128/mra.00411-25

**Published:** 2025-07-23

**Authors:** Mesele Tilahun Belete, Eunhye Park, Younghwan Kim, Seohae Yun, Jong-Ho Lee, Da-Som Lee, Seungmo Lim, Seong-Jin Lee

**Affiliations:** 1 Department of Plant Quarantine, Plant Quarantine Technology Center, Animal and Plant Quarantine Agency 65359, Gimcheon, Republic of Korea; DOE Joint Genome Institute, Berkeley, California, USA

**Keywords:** lychee, HTS, longan witches broom-associated virus, quarantine

## Abstract

The complete sequence of a large single polyprotein of a longan witches’ broom-associated virus (LWBaV) isolate, obtained from post-entry quarantine samples of lychee seedlings from China, was determined by RNA sequencing, RT-PCR amplification, and Sanger sequencing.

## ANNOUNCEMENT

*Litchi chinensis* Sonn., an evergreen fruit tree belonging to the Sapindaceae family, has been cultivated for over 2,300 years in tropical and subtropical regions (1, 2). It is widely grown in Southeast Asia, including Vietnam, China, Thailand, and Cambodia (3). Although no true viruses have been reported in lychee, viroid-like RNA has been detected (2). This study aimed to detect potential viral agents in lychee seedlings imported from China. In the summer of 2024, we collected over 1,000 samples exhibiting symptoms of potential viral infections, including leaf curling, vein clearing, and wilting, at a post-entry quarantine facility in Gimcheon, South Korea. The estimated symptom incidence in lychee seedlings was approximately 10%. Total RNA was extracted from the 1,000 pooled samples using a Pure Strong Plant RNA Extraction Kit (Infusion Tech, Gyeonggi-do, South Korea) (4). After the removal of rRNA using Ribo-zero^TM^ rRNA removal kit (Epicenter, Madison, WI, USA), a cDNA library was generated with TruSeq Stranded Total RNA low-throughput sample prep kit (Illumina, San Diego, CA, USA) (5). High-throughput sequencing was conducted at Macrogen (Seoul, South Korea) on an Illumina NovaSeq 6000 system. The quality of the raw reads was assessed using FastQC (v0.11.7), and Trimmomatic (v0.38) was used to remove low-quality reads and PCR duplicates (5). The 71.1 million high-quality trimmed reads were then *de novo* assembled into 102,976 contigs using Trinity vr20140717, all tools were run with default parameters (5). A BLASTn search was performed against the NCBI nr/nt database (accessed in Feb 2025), identified 14 contigs (217–1,342 nt, average GC:39.84%) associated with the genome sequence of longan witches’ broom-associated virus (LWBaV) isolate Han1 (KY649478) with 93%–100% nucleotide sequence identity.

To confirm the presence of LWBaV, RT-PCR was performed using two published primer sets (3) that target the flanking regions of the NIb-CP and CP genes, successfully amplifying fragments of 1,735 and 617 bp, respectively. Additionally, to determine the complete polyprotein-coding sequence, we designed seven partially overlapping primer pairs (Table 1) that nearly covered the complete polyprotein region based on the reference genome (accession no. KY649478). All amplified PCR products were cloned into RBC-T&A vector and sequenced by Sanger sequencing (5). DNAMAN (v5.2.10) (Lynnon BioSoft, CA, USA) was used to assemble the complete polyprotein sequence, consisting of 9,261 nucleotides, and was deposited in GenBank (accession no. PV470486). The sequence includes cleavage sites, consistent with a previously reported LWBaV isolate from Vietnam, and exhibits 92.3% nucleotide sequence identity with 100% query coverage (accession no. NC_034835).

**TABLE 1:** Primers used for RT-PCR amplification of the longan witches’ broom-associated virus genome

Primer name	Primer sequence 5′→3′	Location	PCR size
LWBaV/F1	GGGTCCATTGAAGTCCCTTT	241–260	1,143
LWBaV/R1	CGGCATGAATGTCTTGATGA	1364–1383	
LWBaV/F2	CTACGTCAATGCGATGTTCG	1165–1184	1,550
LWBaV/R2	CGTGGATCTTGGATTGCTTT	2695–2714	
LWBaV/F3	CTTGGCCTCCTTTGAGAGAA	2432–2451	1,546
LWBaV/R3	CGAGTCGGTTCACAAATCAG	3958–3977	
LWBaV/F4	TCCGGTCATCAAATAGCACA	3860–3879	1,388
LWBaV/R4	AATAGCCTTGTCTGCGTCCA	5228–5248	
LWBaV/F5	TGCCTTACTTCTGTCAACAGGT	5123–5144	1,365
LWBaV/R5	CGACCTGACGCACTTTCAAT	6468–6487	
LWBaV/F6	CTGAATTACAAGGGGATCTTT	6401–6421	1,395
LWBaV/R6	CCATAAACTCCAAGCGTAAC	7776–7795	
LWBaV/F7	ACCACGCCTTCTATTCAGCACATC	7601–7624	1,735
LWBaV/R7	CCTCCCATGGCATCGTTTGTAGTG	9312–9335	
LWBaV-F	AGGGCATTGATCTTGGTGTC	1st detection primer	442
LWBaV-R	CCAGCCACTTCCAGAACTTT		

LWBaV belongs to the *Potyviridae* family, possesses a positive-sense single-stranded RNA genome, and was first identified in a longan plant in Hanoi, Vietnam (3, 6–8). Although the exact cause has not been clearly identified since 1948, studies have suggested an association between filamentous viruses, mites, and phytoplasmas. Surprisingly, LWBaV is considered one of the most destructive pathogens, causing a 10% to 50% reduction in fruit yield (3, 6). As a result, the LWBaV-infected plants used in this study were discarded.

The data provide crucial insights for further investigation into its transmission mechanisms and host range.

## Data Availability

The complete polyprotein sequence of LWBaV has been deposited in GenBank under accession number PV470486. Raw data were deposited in the NCBI Sequence Read Archive (SRA) under accession number PRJNA1256634. The raw Sanger sequencing data files (.abl and. txt format) used for polyprotein assembly have been deposited in Zenodo and are available at https://doi.org//10.5281/zenodo.15571856.
